# Diversity and Inter-Connections in the CXCR4 Chemokine Receptor/Ligand Family: Molecular Perspectives

**DOI:** 10.3389/fimmu.2015.00429

**Published:** 2015-08-21

**Authors:** Lukas Pawig, Christina Klasen, Christian Weber, Jürgen Bernhagen, Heidi Noels

**Affiliations:** ^1^Institute of Molecular Cardiovascular Research (IMCAR), RWTH Aachen University, Aachen, Germany; ^2^Institute of Biochemistry and Molecular Cell Biology, RWTH Aachen University, Aachen, Germany; ^3^Institute for Cardiovascular Prevention (IPEK), Ludwig-Maximilians-University Munich, Munich, Germany; ^4^DZHK (German Centre for Cardiovascular Research), Partner Site Munich Heart Alliance, Munich, Germany; ^5^Cardiovascular Research Institute Maastricht (CARIM), Maastricht University, Maastricht, Netherlands; ^6^August-Lenz-Stiftung, Institute for Cardiovascular Research, Ludwig-Maximilians-University Munich, Munich, Germany

**Keywords:** chemokine, signaling, CXCR4, CXCL12, MIF, CXCR7, ACKR3, ubiquitin

## Abstract

CXCR4 and its ligand CXCL12 mediate the homing of progenitor cells in the bone marrow and their recruitment to sites of injury, as well as affect processes such as cell arrest, survival, and angiogenesis. CXCL12 was long thought to be the sole CXCR4 ligand, but more recently the atypical chemokine macrophage migration inhibitory factor (MIF) was identified as an alternative, non-cognate ligand for CXCR4 and shown to mediate chemotaxis and arrest of CXCR4-expressing T-cells. This has complicated the understanding of CXCR4-mediated signaling and associated biological processes. Compared to CXCL12/CXCR4-induced signaling, only few details are known on MIF/CXCR4-mediated signaling and it remains unclear to which extent MIF and CXCL12 reciprocally influence CXCR4 binding and signaling. Furthermore, the atypical chemokine receptor 3 (ACKR3) (previously CXCR7) has added to the complexity of CXCR4 signaling due to its ability to bind CXCL12 and MIF, and to evoke CXCL12- and MIF-triggered signaling independently of CXCR4. Also, extracellular ubiquitin (eUb) and the viral protein gp120 (HIV) have been reported as CXCR4 ligands, whereas viral chemokine vMIP-II (Herpesvirus) and human β3-defensin (HBD-3) have been identified as CXCR4 antagonists. This review will provide insight into the diversity and inter-connections in the CXCR4 receptor/ligand family. We will discuss signaling pathways initiated by binding of CXCL12 vs. MIF to CXCR4, elaborate on how ACKR3 affects CXCR4 signaling, and summarize biological functions of CXCR4 signaling mediated by CXCL12 or MIF. Also, we will discuss eUb and gp120 as alternative ligands for CXCR4, and describe vMIP-II and HBD-3 as antagonists for CXCR4. Detailed insight into biological effects of CXCR4 signaling und underlying mechanisms, including diversity of CXCR4 ligands and inter-connections with other (chemokine) receptors, is clinically important, as the CXCR4 antagonist AMD3100 has been approved as stem cell mobilizer in specific disease settings.

## Introduction

The CXC chemokine receptor CXCR4 is well known for its role in the homing of progenitor cells into the bone marrow. Upon injury or stress or CXCR4 blockade, progenitor cell mobilization to the periphery is enhanced (1). These functions are based on the role of CXCR4 in mediating the chemotaxis and/or arrest of CXCR4-expressing progenitor cells triggered by the CXCR4 ligand CXCL12, which is secreted by bone marrow stromal cells, but also increased in the periphery at sites of injury or cell stress. Similarly, CXCR4 influences trafficking of other cell types such as lymphocytes and neutrophils, but also CXCR4-positive cancer cells. Based on these functions, CXCR4 has been widely studied in different diseases. For example, blocking the CXCL12/CXCR4 axis with the CXCR4 antagonist AMD3100 or Plerixafor is clinically approved for the mobilization of hematopoietic progenitor cells in combination with granulocyte-colony stimulating factor (G-CSF) in patients with non-Hodgkin’s lymphoma and multiple myeloma (2). Furthermore, CXCR4 has been intensively studied in conditions of injury and ischemia, such as vascular restenosis upon stent implantation or myocardial infarction, respectively, as reviewed in detail recently elsewhere (1). Also, CXCR4 has been under intensive investigation in the area of cancer and different auto-immune diseases such as rheumatoid arthritis and systemic lupus erythematosus (2, 3). Mostly, these roles of CXCR4 have been linked to its capacity to bind its cognate ligand CXCL12.

However, it has become clear that CXCR4-mediated signaling is more complex as initially thought, as also macrophage migration inhibitory factor (MIF) and extracellular ubiquitin (eUb) have been shown to bind CXCR4 and induce intracellular signaling (4, 5). Furthermore, the chemokine receptor ACKR3 (previously CXCR7) has added to the complexity of CXCR4 signaling due to its ability to bind not only CXCL11 but also CXCL12 and to interact with MIF, regulating ligand availability for CXCR4 and evoking CXCL12- and MIF-triggered signaling independently from CXCR4 (6, 7). Also, the endogenous, antimicrobial protein human β3-defensin (HBD-3) was identified as a novel antagonist of CXCR4, blocking CXCL12-induced CXCR4 signaling (8). Moreover, CXCR4 binds the exogenous HIV protein gp120, and is therefore crucially involved in HIV infection (2, 9). On the other hand, CXCR4 is antagonized by the viral macrophage inflammatory protein-II (vMIP-II). vMIP-II is a CC chemokine expressed by Kaposi’s sarcoma-associated herpesvirus, functions as an antagonist for multiple chemokine receptors, and is employed by the virus to escape the host immune system (10, 11).

Here, we will summarize the structural characteristics of the chemokine receptor CXCR4, its chemokine ligands CXCL12 and MIF, as well as non-canonical and non-host ligands eUb and HIV gp120. We will review and discuss current knowledge on intracellular signaling through CXCR4 and biological effects triggered by each of these CXCR4 ligands, and will elaborate on how ACKR3 modulates classical CXCR4 signaling triggered by CXCL12. Also, we will discuss the antimicrobial protein HBD-3 as well as the viral chemokine vMIP-II as an endogenous, respectively, viral antagonist for CXCR4 (8, 10), summarize the efforts that have been undertaken in developing CXCR4 antagonists for clinical use, and summarize clinical trials that are currently ongoing and that target CXCR4 in different pathologies. Getting insight into the signaling mechanisms of CXCR4 by different ligands, modulation by CXCR4-containing receptor complexes and resulting biological effects is important to understand the potential merits of CXCR4 inhibition in the context of different pathologies, but also to be able to foresee potential side effects of long-term interference with CXCR4.

## Structural Characteristics Affecting CXCR4 Functional Properties

### The chemokine receptor CXCR4

Chemokines carry conserved cysteine motifs and are defined by those cysteine “signature motifs.” This has led to a categorization into four classes of chemokines, i.e., the CXC, CC, C, or CX_3_C chemokines where C is a cysteine residue and X any amino acid (aa) residue (12) (Box 1). Chemokine receptors represent a sub-class of G protein-coupled receptors (GPCRs). Since chemokine receptors are classified according to the respective ligand classes, CXCR4 belongs to the family of CXC chemokine receptors (12, 13). Historically, CXCR4 was termed leukocyte-derived seven-transmembrane domain receptor (LESTR) following its cloning from a cDNA library of human monocytes in 1994 (14). Alternative names were cluster of differentiation 184 (CD184) and fusin, the latter defined as a co-factor for the fusion and entry of HIV-1 (15). The term CXCR4 was proposed by Oberlin et al. in 1996, who were the first to describe CXCL12 as a ligand for this receptor (16).

Box 1 **Chemokines and chemokine receptors.**

Chemokines are 8–10 kDa small proteins that belong to the superfamily of cytokines and induce chemotactic cell migration by binding to their corresponding receptors. In this way, chemokines mediate the formation of tissues, e.g., during embryogenesis or wound healing, as well as the recruitment of immune cells out of the bloodstream to sites of injury and infection (17). Chemokines are classified into four conventional subgroups according to the number and positioning of certain conserved N-terminal cysteine residues. The C-chemokines exhibit only one N-terminal cysteine, while the CC-family has two cysteines localized side by side. In the CXC chemokine family, the two cysteines are spaced from each other by one amino acid, whereas in CX_3_C chemokines they are separated by three amino acids (12, 18). Additionally, a family of chemokine-like function (CLF) chemokines was defined to exhibit typical chemokine activities in the absence of a prototypical N-terminal cysteine motif (19).

Chemokines mediate their effects by binding to corresponding chemokine receptors, whose nomenclature aligns with their ligands, i.e., CR, CCR, CXCR, and CX_3_CR. More generally, chemokine receptors are divided into two groups: the GPCRs, which signal via G proteins and induce various cellular functions including cell migration or leukocyte arrest, and the atypical chemokine receptors (ACKRs), which are structurally homologous to the GPCRs but are unable to couple to G proteins due to a lacking cytosolic DRYLAIV motif, and fail to induce classical chemokine signaling. Because ACKRs efficiently internalize their ligands, they can function, on the one hand, as chemokine decoy receptors and, on the other hand, they concentrate chemokines in hard-to-reach domains (20). For example, chemokine transcytosis across biological barriers mediated by the ACKR “DARC” on venular endothelial cells was shown to result in apical retention of chemokines and enhanced leukocyte migration across DARC-expressing monolayers (21).

Some chemokines exclusively bind to one receptor, while others bind a variety of receptors. Inversely, some chemokine receptors only bind one ligand, while others bind several chemokines (12, 18). This variability is referred to as “redundancy” or “promiscuity.” In addition, chemokine receptor expression varies between different cell types. This capacious complexity of the chemokine/chemokine receptor network enables the fine-tuned recruitment of defined cell types to their place of destination (18).

**Figure 1:**
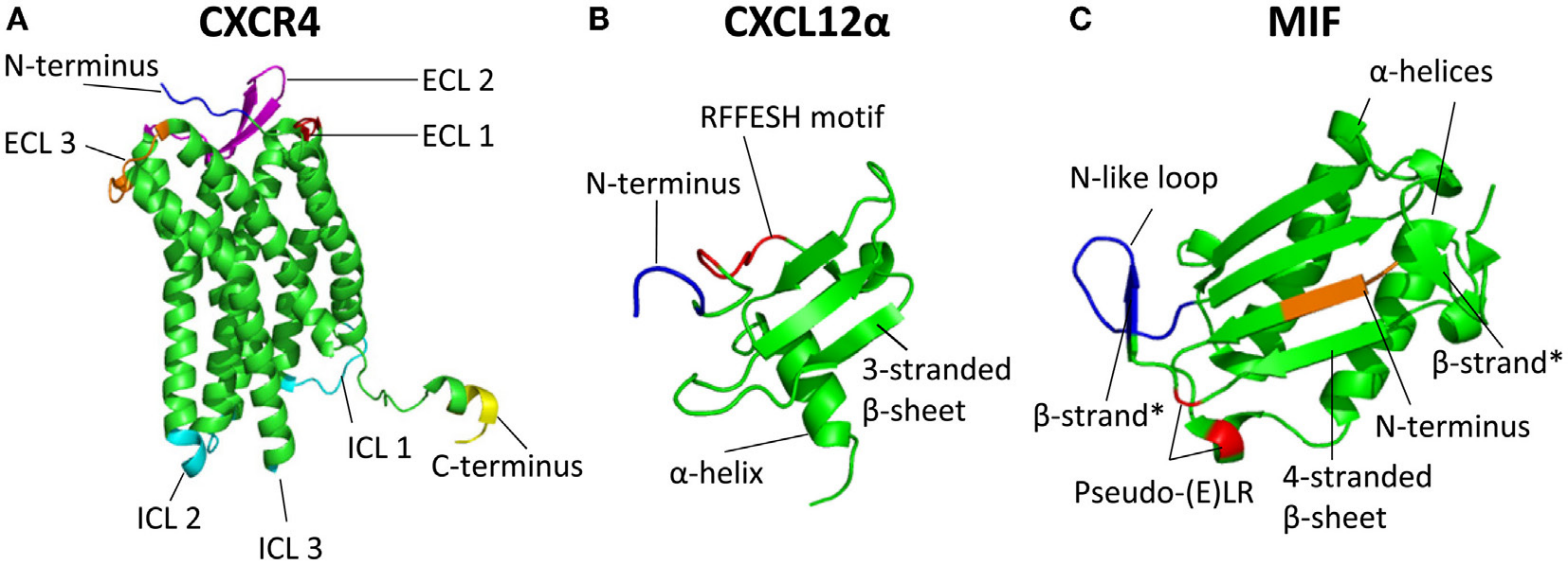
**Three-dimensional structure of the chemokine receptor CXCR4 and its ligands CXCL12 and MIF**. **(A)** Crystal structure of CXCR4 [Protein data bank identifier 3ODU; (24)]. Included in the crystal structure are amino acids (aa) 27 to 319 of the 352 residues of CXCR4. CXCR4 can exist as a homodimer; here, only chain A is depicted. The N-terminal aa 27–31 are depicted in blue, extracellular loop 1 (aa 100–104) in red, extracellular loop 2 (aa 174–192) in purple, and extracellular loop 3 (aa 267–273) in orange. Intracellular loops (ICL) 1 (aa 65–71), ICL2 (aa 140–144), and ICL3 (aa 225–230) are depicted in cyan. The C-terminal aa 315–319 are shown in yellow. The degradation motif SSLKILSKGK (aa 324–333) cannot be depicted here due to the shortened sequence in the crystal structure. **(B)** Crystal structure of CXCL12 isoform α [Protein data bank identifier 3GV3; (26)]. Included in the crystal structure are aa 4–67 (with aa counting referring to the mature CXCL12). Here, a CXCL12 monomer is depicted with the N-terminal aa 4–7 in blue. The RFFESH motif (aa 12–17), which is the most important binding motif for CXCR4 in the CXCL12 core, is depicted in red. Each monomer includes a three-stranded β-sheet and one α-helix. **(C)** Crystal structure of MIF [Protein data bank identifier 3DJH; (27)]. Included in the crystal structure are aa 2–114 (with aa counting including Met-1). A MIF monomer is depicted. The N-terminal aa 2–5 are shown in orange. Depicted in red is the pseudo-(E)LR motif (Asp45–X–Arg12), the N-like loop (aa 48–57) is depicted in blue. Each monomer includes two antiparallel α-helices and a four-stranded β-sheet. Two additional, short β-strands (aa 48–50 and aa 107–109) can be found in each monomer stabilized by trimer formation, and are indicated with “β-strand*.”

CXCR4 is an evolutionary conserved protein with 89% similarity between the human [352 aa] and mouse (359 aa) ortholog (22). Ubiquitous expression of CXCR4 has been detected in bone marrow, lymph nodes, liver, lung, brain, heart, kidney, thymus, stomach, pancreas, spleen, ovary, and small intestine (23). Similar to other GPCRs, CXCR4 has an extracellular N-terminus (34 aa for CXCR4), seven transmembrane alpha helices connected by three extracellular and three intracellular loops (ICL), and a C-terminus that is located in the cytoplasm (Figure 1A). However, analysis of crystal structures of CXCR4 in complex with the small-molecular antagonist IT1t and the cyclic peptide antagonist CVX15 in 2010 revealed specific differences in the orientation of the alpha helices compared with other available GPCR structures, mostly at the extracellular side (24). One important difference is the extension of the extracellular end of helix VII, which is two helical turns longer as in other GPCR structures and allows a disulfide bond of Cys274 in helix VII with Cys28 in the N-terminal region. This disulfide bond, together with and among chemokine receptors highly conserved disulfide bond between extracellular loop 2 (ECL2, Cys186) and the extracellular end of alpha helix III (Cys109), is essential for CXCL12 binding by shaping the entrance to the ligand-binding pocket (24). Compared to the extracellular half of CXCR4, which overall substantially differs from other GPCRs, the intracellular part of CXCR4 is structurally fairly similar to the intracellular half of other GPCRs. As exception, the intracellular part of helix VII of CXCR4 is one turn shorter compared to other available GPCR structures. Furthermore, the CXCR4 crystal structure lacks the short alpha helix VIII present in many GPCRs and also lacks in its C-terminus a palmitoylation site, which in many GPCRs attaches to the lipid membrane (24). Crystallization studies revealed CXCR4 to exist as a homodimer, however, left open the possibility of CXCL12/CXCR4 binding in a 1:1 or 1:2 ligand:receptor stoichiometry (24). Recently, a combination of computational, functional, as well as biophysical methods supported a 1:1 over a 1:2 CXCL12:CXCR4 binding stoichiometry (25). This was recently further supported by the crystal structure of CXCR4 in complex with the high-affinity antagonist vMIP-II encoded by Kaposi’s sarcoma-associated herpesvirus (as discussed in more detail later). CXCR4 interacted as a dimer with vMIP-II in a 1:1 stoichiometry (11). In addition to homodimerization, CXCR4 can also form dimers with ACKR3 and other receptors, as discussed in detail later.

Upon ligand binding, CXCR4 is internalized by endocytosis and degraded in lysosomes, mediated by a degradation motif (SSLKILSKGK) in its C-terminus and ubiquitination of vicinal lysine residues (28).

### Endogenous ligands of CXCR4: CXCL12, MIF, and eUb

#### CXCL12

The chemokine CXCL12 or stromal cell-derived factor-1 (SDF-1), which is the best known ligand of CXCR4, i.e., the “cognate” ligand of CXCR4, is highly conserved between mouse and human (>92%), suggesting an evolutionarily important role (29). Six isoforms of human CXCL12 have been defined, with CXCL12-α and CXCL12-β representing the major (“classical”) isoforms, and CXCL12-γ, CXCL12-δ, CXCL12-ε, and CXCL12-φ being less well characterized. CXCL12 isoforms are generated by differential splicing events. All isoforms share the first three exons, with observed differences in the fourth exon only. The CXCL12-α pro-protein (before N-terminal processing and secretion) is an 89 aa protein, while the other isoforms have extensions at the C-terminus with an additional 4 aa (CXCL12-β), 20 aa (CXCL12-γ), 51 aa (CXCL12-δ), 1 aa (CXCL12-ε), and 11 aa (CXCL12-φ) (1, 30). Not all isoforms show an identical expression pattern, with CXCL12-γ in adult mice mainly expressed in brain, heart, and the endothelium of vessels, compared to a broader expression pattern of CXCL12-α (31). On functional level, most research has focused on CXCL12-α, and the isoforms CXCL12-β and even to a higher extent -γ, -δ, -ε, and -φ have still been poorly characterized. However, functional differences have been revealed between the different isoforms, with, for example, CXCL12-γ binding the receptor CXCR4 with only low affinity and triggering reduced chemotaxis *in vitro* compared to CXCL12-α (31, 32). Also, the γ isoform has been shown to be present in the nucleus of mouse cardiac tissue by transcription of a distinct mRNA lacking the N-terminal signal peptide responsible for chemokine secretion (as explained in more detail below), suggesting specific intracellular functions different from the extracellular functions of the α and β isoforms (33). Furthermore, an isoform-specific role of CXCL12 has previously been suggested in the context of cerebral ischemia, where leukocyte infiltration was associated with endothelial CXCL12-β but not −α (34). In comparison to the human system, there are only three CXCL12 isoforms described in mouse. These are Cxcl12-α, -β, and -γ, which correspond to the respective human isoform counterparts, with only a single homologous aa substitution (Val to Ile substitution at aa 18 in the mature CXCL12 protein) from human to mouse (32, 33, 35).

The CXCL12 “pro-protein” contains a signal peptide of 21 aa at the CXCL12 N-terminus, which is cleaved off before secretion of the mature, biologically active CXCL12 protein. In the literature, residue numbers of important motifs of CXCL12 are numbered starting from Lys-22 in the pro-protein, now being counted as Lys-1 in the mature protein. The CXCL12 residue numbers mentioned in this manuscript are numbered accordingly.

The structure of the mature CXCL12 protein is characterized by a three-stranded β-sheet that is packed against an α-helix (Figure 1B) and extends to a six-stranded β-sheet in dimeric CXCL12 species (see below). The N-terminus of mature CXCL12, in particular the first two residues Lys-1 and Pro-2 (with aa indication referring to their position in mature CXCL12 throughout this manuscript), is essential for CXCR4 activation, as shown by the observation that loss of these first two residues completely abolished CXCR4 activation, while CXCR4 binding affinity was decreased 10-fold (36). A report by Crump et al. (36) and subsequent studies (18) support a so-called “two-site” model of chemokine binding to their receptors: “Site one” consists of the chemokine core domain and is responsible for docking of the chemokine to its receptor. In CXCL12, the most important core domain for CXCR4 binding is the so-called RFFESH motif (residues 12–17 in mature CXCL12). “Site two” consists of the N-terminus of CXCL12, more precisely especially Lys-1 and Pro-2, which activate CXCR4 signaling (36). The differential C-termini in the different CXCL12 isoforms are not involved in either of these site one or two interactions with CXCR4.

This “two-site” model has been proposed as a general functional mechanism of chemokines for a long time (18, 37). However, which residues of CXCR4 in particular are involved in site one and site two interaction with CXCL12 still remains to be elucidated in more detail. An important contribution to CXCL12 binding was revealed to occur through posttranslational sulfation of tyrosine residues in the CXCR4 N-terminus (Tyr-21, Tyr-12, Tyr-7). This increases the binding affinity of CXCR4 for CXCL12 through electrostatic interactions between acidic sulfated tyrosines within CXCR4 and basic residues within CXCL12 (38, 39) and is expected to contribute to “site one” interaction between CXCR4 and CXCL12. More specifically, sulfated Tyr-21 was recently predicted to interact with the N-loop-β1 strand junction within CXCL12 based on the crystal structure of CXCR4 bound to the viral chemokine vMIP-II (11). Furthermore, this latter study revealed within the β3 strand of vMIP-II a stretch of four residues (RQVC, aa 48–51) that interacted with CXCR4 residues D22 and E26 and that was previously shown to be important for interaction of CXCR4 with vMIP-II (11). These same residues RQVC are conserved in CXCL12 (aa 47–50), also previously shown to be important for CXCL12/CXCR4 binding, and structural modeling predicted their involvement in CXCL12/CXCR4 interaction comparably to vMIP-II/CXCR4 interaction (11). Furthermore, the crystal structure of CXCR4 in complex with IT1t as competitive antagonist of CXCL12 revealed as important ligand/receptor contact points the acidic residues Asp-187, Glu-288, and Asp-97 in CXCR4, which were previously also shown to be important for CXCL12 binding (24). With respect to the “site two” interaction important for receptor signaling, the crystal structure of CXCR4 in complex with the antagonistic peptide CVX15 was suggested to reveal a “site two” interaction of the CXCL12 N-terminus (aa 1–8 KPVSLSYR) with CXCR4, with preliminary modeling studies suggesting that CXCL12 Lys-1 could integrate into the CXCR4 binding pocket and interact with available acidic aa (24). Together, these findings underline the notion that important insights into the interaction of CXCR4 with its ligand CXCL12 can be deduced from available crystal structures of CXCR4 with other ligands or small-molecule antagonists.

Mature CXCL12 is intrinsically unstable and truncated variants of mature CXCL12 missing 2,3,5 or 7 N-terminal aa have been identified in human blood, with only non-truncated CXCL12 able to induce intracellular calcium flux and chemotaxis of stem cells *in vitro* (40). Proteases able to inactivate CXCL12 by cleaving off N-terminal residues from CXCL12 *in vitro* include dipeptidyl peptidase 4 (DPP4, also known as CD26), matrix metalloproteinase 2, neutrophil elastase, and cathepsin G (Box 2). For example, CXCL12 has a half-life of less than 1 min for truncation by DPP4 *in vitro* (41). This intrinsic instability of CXCL12 could at least partly contribute to the short life-time of CXCL12 *in vivo*, with wild-type CXCL12 effectively cleared from mouse blood within 1 h of intravenous application (42).

Box 2 **Instability of CXCL12.**

Signaling of the CXCL12/CXCR4 axis is tightly regulated. One way how signaling can be terminated is by proteolytic cleavage of CXCL12. Particularly interesting is dipeptidyl peptidase IV (DPP4, also known as CD26), an enzyme that cleaves off the first two residues of CXCL12 (43), as also shown in human serum (44). Removal of these first two amino acids is sufficient to completely abolish CXCL12-induced CXCR4 activation despite an only 10-fold reduction in CXCR4 binding (36). DPP4, which can be membrane-expressed in addition to its soluble form, also co-localizes and co-immunoprecipitates with CXCR4 (45), which further establishes a specific role for this protease in the regulation of CXCR4 signaling.

Furthermore, matrix metalloproteinase 2 (MMP-2) is capable of cleaving the first four N-terminal amino acids of CXCL12, and was shown to impair CXCR4 signaling in the context of recruitment of neural progenitor cells (46). MMP-2 was first shown to inactivate CXCL12 in *in vitro* experiments by McQuibban et al., alongside the MMP family members MMP 1, 3, 9, 13, and 14 (47). Also, neutrophil elastase, released from mononucleated blood cells or polymorphonuclear neutrophils, can cleave the first three N-terminal residues of CXCL12 inducing its inactivation. In addition, it can cleave the N-terminus of CXCR4, which significantly reduces CXCR4 binding to CXCL12 (48). Finally, cathepsin G has been shown to inactivate CXCL12 by cleaving the first five residues at the N-terminus (49).

In addition, fast clearance of CXCL12 from blood seems to be partially mediated by sequestration of CXCL12 to heparan sulfate on the surface of endothelial cells through the BBXB heparin sulfate-binding motif (aa) KHLK in position 24–27; with B = basic aa and X = any other aa) in the first strand of the β-sheet of CXCL12 (50): a CXCL12 mutant that failed to bind heparan sulfate proteoglycans was retained up to 60% in the blood of mice 6 h after injection (42). Of note, the C-terminus of the CXCL12-γ isoform encompasses an additional four overlapping BBXB heparan sulfate-binding motifs, which mediate strong absorption of CXCL12-γ on the plasma membrane after secretion through interaction with cell membrane glycosaminoglycans. This enables for efficient formation of chemokine gradients (“haptotactic gradients”) directing cell migration and angiogenesis *in vivo* with higher efficiency compared to CXCL12-α, despite a lower binding affinity to CXCR4 (31). Although also the CXCL12-β isoform contains one additional BBXB motif in its C-terminus, it does not show higher affinity to heparin sulfate compared to the α-isoform. Interestingly, a knock-in mouse line carrying a mutated Cxcl12 that cannot bind heparin sulfate without effect on Cxcr4 signaling by the α, β, or γ isoform showed an increased number of circulating hematopoietic progenitor cells, but a reduction in the number of cells infiltrating ischemic tissue after acute hindlimb ischemia and associated revascularization (51). On the other hand, a recent report revealed truncation of five or seven N-terminal residues from CXCL12 to increase its binding affinity to the glycosaminoglycan heparin. As both truncated variants were able to induce stem cell mobilization in mice although they displayed *in vitro* no or a negative effect on CXCL12-induced chemotaxis, respectively, these findings may suggest that N-terminally truncated CXCL12 variants may influence stem cell mobilization by regulating binding of CXCL12 to glycosaminoglycans rather than to CXCR4 (40). Together, these findings reveal an important role for glycosaminoglycans in regulating CXCL12 functions.

Finally, *in vitro* experiments indicated that CXCL12 may exist in both monomeric and dimeric forms in the extracellular space. Although recent findings support a 1:1 over a 1:2 ligand:receptor stoichiometry for interaction of CXCR4 with both CXCL12 (25) as well as vMIP-II (11), it remains unclear whether dimeric CXCR4, as revealed in crystal structures, would preferentially bind dimeric CXCL12 or rather two monomeric CXCL12 proteins (24). Of note, distinct CXCL12 oligomers have been associated with differential downstream signaling, although with contradictory findings, as discussed in more detail later.

#### Migration inhibitory factor

Migration inhibitory factor is a 12.3 kDa small cytokine with chemokine-like properties. It is quasi-ubiquitously expressed in various tissues in mammals and its structure is highly conserved with about 90% sequence homology between mouse and human species (52). MIF consists of 115 aa, but the N-terminal methionine residue is posttranslationally removed after ribosomal synthesis in essentially all cells and organisms. Crystallographic studies revealed MIF as a homotrimer consisting of three monomers that each has two antiparallel α-helices and a four-stranded β-sheet. Two additional, short β-strands can be detected in each monomer which interact with the β-sheet of the adjacent subunits (53) (Figure 1C). However, different studies revealed MIF to be able to exist as monomer, dimer, trimer, or even higher-order oligomers, potentially concentration-dependent, although the biologically relevant “active” oligomerization state of MIF is still elusive (53–55). As interference with MIF trimerization using the inhibitor ebselen increased MIF’s chemotactic capacity, it is however likely that the MIF homotrimer is not the only biologically active form (56). For example, MIF was recently identified as a chaperone molecule inhibiting the accumulation and mitochondrial association of misfolded superoxide dismutase SOD1, and gel filtration fractions of cellular lysates containing this inhibitory potential contained monomeric as well as oligomerized MIF (57). Of note, a chaperone function of MIF was recently suggested toward insulin to ensure full insulin function through effects on insulin conformation (58).

Migration inhibitory factor lacks a typical N-terminal leader sequence indicating that it is secreted by a non-classical secretion pathway (59). Because MIF is missing an N-terminal cysteine motif, it cannot be grouped into one of the four classical chemokine groups (C, CC, CXC, and CX_3_C), which are classified by the presence and spacing of their N-terminal cysteine residues (Box 1). Nevertheless, MIF exhibits potent chemotactic properties through interaction with classical chemokine receptors and thus is a protagonistic member of the group of CLF chemokines (19, 60). MIF mediates its chemotactic effects by binding to the chemokine receptors CXCR2 and CXCR4 (4), as discussed in more detail below. MIF exhibits a structural motif (Asp45–X–Arg12, “DXR,” with aa numbering for MIF including Met-1 throughout this manuscript) similar to the conserved N-terminal ELR (Glu–Leu–Arg) motif of the cognate CXCR2 ligands, which is important for their efficient binding to CXCR2. In MIF’s so-called “pseudo-(E)LR motif,” the glutamate (Glu) is exchanged by an aspartic acid (Asp), representing a conservative substitution. Furthermore, the Asp and Arg residues in this motif are located in neighboring loops in 3D space, but show similar spacing as the authentic ELR motif (61). Mutations of Asp45 or Arg12 indeed abrogated MIF/CXCR2-mediated effects indicating the “pseudo-(E)LR motif” as structural determinant for MIF binding to CXCR2 (61). Additionally, an N-like loop including the aa from position 48 to 57 of the MIF protein was identified as being important for MIF-CXCR2 binding (62). These findings suggest a two-site binding mechanism for MIF-CXCR2 interaction that is reminiscent of that of cognate CXCR2 ligands. Site one, consisting of the N-like loop and the CXCR2 N-domain, supposedly initializes the interaction, while the site two interaction between the pseudo-(E)LR motif and the extracellular loops of CXCR2 is assumed to lead to receptor activation (63). The interaction sites between MIF and CXCR4 still need to be identified. Additional structural characteristics of MIF related to its intrinsic catalytic oxidoreductase and tautomerase activities were recently discussed at length (19, 64) and will not be further reflected herein.

#### Extracellular Ubiquitin (eUb)

Ubiquitin is a small 8.6 kDa protein constitutively expressed in all eukaryotic cells which displays a very conserved structure with only little variance from insects to humans (65). Its tightly controlled expression is encoded by four genes in mammals (66). Because its amino acid structure includes seven lysines, ubiquitin can build poly-chains (67). Therefore, intracellular ubiquitin can appear as a free monomer, a free polyubiquitin chain, or as a monomer or a polyubiquitin chain conjugated to a substrate (66). Ubiquitin is well characterized as a posttranslational protein modifier, with Lys-48-linked polyubiquitin chains binding covalently to lysine residues of target proteins to induce their degradation through the 26S ubiquitin-proteasome degradation pathway. However, ubiquitin also serves different degradation-independent functions affecting intracellular processes in a reversible way, as, for example, in regulating protein activity, subcellular localization, and interaction with other proteins. This is mediated by a structurally different ubiquitination of target proteins, involving the coupling of target proteins to ubiquitin monomers or polyubiquitin chains interlinked through ubiquitin lysines other than Lys-48 (e.g., Lys-63) [reviewed in Ref. (65)].

But ubiquitin is not only an intracellular protein modifier, but it is also a natural plasma protein and can be detected in urine (68). Nevertheless, not much attention has been paid to ubiquitin’s “extracellular” actions. Although the existence of a cell surface receptor for ubiquitin was assumed since its discovery in 1975 (69), first evidence that CXCR4 may function as a cell surface receptor for ubiquitin was only revealed in 2010 upon studying the effect of CXCR4 antagonism or knockdown on binding of ubiquitin to CXCR4-positive cells (70). Studies concerning the structural determinants of the ubiquitin–CXCR4 interaction revealed by binding assays using fluorescently labeled ubiquitin indicated a two-site binding mechanism involving the flexible C-terminus of ubiquitin and its hydrophobic surface surrounding Phe-4 and Val-70. Within CXCR4, residues Phe-29, Phe-189, and Lys-271, which do not contribute to the CXCL12–CXCR4 interaction interface, seem to be important for ubiquitin-CXCR4 binding (71).

### Gp120 as exogenous ligand of CXCR4

The HI-virus (HIV) is surrounded by an envelope consisting of a host cell-derived lipid bilayer and virus-encoded glycoproteins. To enter a new target cell, the virus membrane has to be fused with the target cell membrane, a process mediated by the virus glycoprotein gp120 (Box 3) (72). Gp120 has a molecular weight of 120 kDa. Its secondary structure involves four surface-exposed loops which are formed by variable regions (V1–V4) with disulfide bonds at their bases. They are divided by five relatively conserved domains (C1–C5) (73). The envelope glycoproteins of HIV are encoded by the virus RNA *env*-gene (74). They are synthesized as precursors in the endoplasmic reticulum of the infected cell. After addition of asparagine-linked high-mannose sugar chains, the resulting glycoprotein gp160 is transported to the Golgi apparatus, where it is cleaved by cellular proteases into the functional envelope proteins gp120 and gp41 (75, 76). The mature glycoproteins gp41 and gp120 build up complexes which are translocated to the cell surface, where they are integrated into sprouting virions (77). Gp41 functions as a transmembrane protein and gp120 is the exterior envelope protein capping gp41 (78). The evolving so-called Env protein, a trimer of gp41–gp120 heterodimers, mediates target cell receptor binding (79). First, the CD4 binding site of gp120, consisting of the hydrophobic regions around Thr-257 and Trp-427 and the hydrophilic regions around Asp-368 and Glu-370, binds to CD4 (80). This binding induces rearrangement of V1 and V2 and subsequent of V3 (81), enabling binding of V3 to the co-receptor CXCR4 or CCR5 depending on the sequence of V3 (82). Gp120 binding to the chemokine receptors CXCR4 or CCR5 is thereby supposed to be the trigger for membrane fusion of the virus and the target cell, which is responsible for HIV infection (79). The mechanism of binding to chemokine receptors without exhibiting typical chemokine structures, like gp120 binding to CXCR4 or CCR5, is called chemokine mimicry. It is used by several viruses like pox or herpes viruses to strengthen their propagation by blocking chemokine action or triggering chemokine receptor signaling (83). Molecular mechanisms underlying gp120/CXCR4-induced cell death are discussed in more detail below.

Box 3 **Gp120.**

Gp120 is the envelope protein of the HI-virus, which is responsible for the entry of the virus into cells (79). Hence, it is a main virulence factor of HIV. The position of gp120 outside the virus membrane, bound to another envelope protein gp41, enables binding to the CD4 receptor of the target cell (77). After subsequent structural rearrangement of gp120, it can bind to one of the HIV co-receptors, CXCR4 or CCR5, via chemokine mimicry (83). This is the crucial step leading to membrane fusion and consolidation of the virus with its target cell, thereby enabling infection (79). Furthermore, gp120 induces cellular apoptosis, partly through CXCR4. Molecular mechanisms underlying gp120/CXCR4-mediated apoptosis are discussed in more detail elsewhere in this review.

### vMIP-II and human β3-defensin as potent CXCR4 antagonists

#### vMIP-II as Viral CXCR4 Antagonist

Chemokine mimicry, as described for gp120, is also involved in the interaction of CXCR4 with vMIP-II. vMIP-II is a chemokine-like protein encoded by the Kaposi’s sarcoma-associated herpesvirus 8, with about 40% similarity to mammalian chemokines (10). It was described as a potent antagonist for several CC and CXC chemokines and their receptors, including CXCR4, for which it competes with CXCL12 for receptor binding. However, in contrast to CXCL12, vMIP-II cannot induce Ca^2+^ mobilization from intracellular stores, nor induce chemotaxis of human monocytes (10). Zhou et al. revealed in 2000 that the N-terminus of vMIP-II, particularly the first five residues, is essential for binding to CXCR4 (84). Interestingly, native vMIP-II was able to inhibit HIV-1 viral entry into a CD4-expressing U87 cell line *in vitro* by antagonizing CCR3, CCR5 as well as CXCR4 (10). A peptide corresponding to residues 1−21 of vMIP-II showed inhibition of HIV-1 gp120-mediated cell−cell fusion only via CXCR4 (84). Together, these findings implicated vMIP-II as a promising lead molecule for anti-HIV drug development. vMIP-II-derived peptides are also being investigated as CXCR4 inhibitors in the context of cancer therapy (85).

#### HBD-3 as Endogenous CXCR4 Antagonist

Human β3-defensin was proposed as a novel, endogenous antagonist of CXCR4 in 2006 by Feng et al. (8). The β-defensins are a group of antimicrobial peptides present on mucosal epithelium, which are also upregulated in the presence of HIV-1 and block HIV replication by direct binding of virions and blocking of the HIV co-receptor CXCR4 (8). HBD-3 competes with CXCL12 for CXCR4 binding, as shown by the inhibition of CXCL12-induced T-cell chemotaxis, ERK1/2 activation, and Ca^2+^ mobilization upon HBD-3 treatment (8). Confocal microscopy showed that HBD-3 treatment induced internalization of CXCR4 in a T-cell line, however HBD-3 did not trigger downstream signaling such as Ca^2+^ mobilization or ERK phosphorylation, nor chemotaxis (8). The structural features of HBD-3 important in CXCR4 antagonism were unraveled by the same group in 2013, partly by structural comparison with CXCL12. HBD-3 is a protein of 45 aa, with six conserved cysteine residues (Cys-11, 18, 23, 31, 40, and 41) forming three disulfide bonds, which stabilize the protein (86, 87). Substituting these cysteine residues with uncharged aa, generating a “linearized” HBD-3, completely abrogated the inhibitory effect of HBD-3 on CXCL12-triggered Ca^2+^mobilization (87). Similar effects were observed upon substituting the cationic residues Lys8, Lys32, and Arg36, resembling the residues Lys-1, Arg8, and Lys-12 in CXCL12, with neutral ones; substituting the positively charged C-terminus with negatively charged residues; as well as removing the first 10 N-terminal residues (87). Of note, such structural insights into the CXCR4 antagonism by HBD-3 could stimulate the development of novel CXCR4 antagonists.

## Biological Processes and Signaling Triggered by CXCL12 through CXCR4

The CXCL12/CXCR4 axis is involved in a plethora of biological processes, including, for example, progenitor cell homing and mobilization, neutrophil homeostasis, embryonic development, and angiogenesis. These biological effects are mediated by complex signaling mechanisms, including classical GPCR signaling, β-arrestin recruitment, or the activation of the JAK/signal transducer and activator of transcription (STAT) pathway. Also, oligomerization of CXCR4 or CXCL12, as well as interaction of CXCR4 with other signaling molecules affects the outcome of CXCL12/CXCR4 signaling. Each of these aspects will be discussed in the following section.

### Biological processes regulated by the CXCL12/CXCR4 axis

The CXCL12/CXCR4 axis plays an important role in the homing of hematopoietic stem and progenitor cells (HSPCs) in the bone marrow, and their mobilization to the periphery in conditions of stress or injury. Homing of HSPCs is mediated by CXCL12 secretion by endothelial cells in the bone marrow sinusoids as well as by bone marrow stromal cells. CXCR4-positive HSPCs flowing through the bloodstream are triggered to firmly adhere to the endothelium of bone marrow sinusoids through CXCL12/CXCR4-induced integrin activation (88), followed by their migration into specialized bone marrow niches. Constant trafficking of a small number of HSPCs from the bone marrow to the periphery occurs in normal physiological state, however in certain conditions such as injury or stress, an increased release of HSPCs into the bloodstream can be observed. Since in turn CXCL12/CXCR4 signaling is essential for bone marrow retention of HSPCs, interference with this pathway was shown to trigger HSPC mobilization (89). There are several proposed mechanisms of CXCR4 interference in the context of HSPC mobilization, as discussed in more detail recently (1). For example, the commonly used stem cell mobilizing reagent G-CSF was shown to induce the cleavage of the CXCR4 N-terminus on HSPCs, leading to diminished chemotaxis and arrest, and reduced retention of HSPCs in the bone marrow (90). *In vitro*, such CXCR4 cleavage could be induced by the neutrophil proteases neutrophil elastin and cathepsin G (90), although the *in vivo* relevance requires further validation.

In addition to HSPC homeostasis, the CXCL12/CXCR4 axis has been implicated to be involved in progenitor cell survival and proliferation (91, 92). Also, similar to its effect on HSPCs, CXCL12/CXCR4 signaling regulates the homeostasis of CXCR4-positive neutrophils and mediates their homing in the bone marrow through the constitutive expression of CXCL12 by bone marrow stromal cells (93). The retention of neutrophils in the bone marrow by CXCL12/CXCR4 can be blocked by the CXCR4 antagonist AMD3100 (Plerixafor), which has been used to correct neutropenia in patients (94).

Since CXCR4 is crucial in stem cell homeostasis, it is not surprising that it has essential functions in embryonic development. In fact, knockout of *Cxcr4* in mice causes several lethal birth defects, such as defective trafficking of HSPCs from the fetal liver to the embryonic bone marrow, impaired B-lymphopoiesis, impaired vascularization, and abnormal cerebellum development (95, 96). In addition to its role in vascularization during development, the CXCL12/CXCR4 also plays an important role in angiogenesis in the context of ischemia, as discussed in more detail recently (1). CXCL12 expression was shown to be upregulated in conditions of hypoxia in a HIF-1-dependent way, resulting in increased chemotaxis and adhesion of CXCR4-positive progenitor cells to ischemic tissue (97). In a mouse model of myocardial ischemia, CXCL12 treatment leads to increased levels of vascular endothelial growth factor (VEGF) and enhanced neo-angiogenesis, associated with reduced infarction size after myocardial infarction (98). Also, the transplantation of CXCL12-overexpressing endothelial progenitor cells during myocardial infarction in rats could increase neo-angiogenesis (99). In ischemic preconditioning, which exerts cardioprotective effects, the levels of *Cxcr4* mRNA were increased in cardiac myocytes and fibroblasts, suggesting also a local protective effect of the CXCL12/CXCR4 axis. Indeed, the *in vivo* administration of CXCL12 leads to a decrease in myocardial infarct size associated with signaling through the anti-apoptotic kinases ERK 1/2 and AKT in cardiac cells (99).

Related to its role in angiogenesis, chemotaxis (100, 101), and cell proliferation (100, 102), CXCL12/CXCR4 signaling has also been linked to different pathologies including tumor progression and metastasis, as discussed in more detail later.

### CXCL12/CXCR4 binding triggers G protein-coupled signaling

**Figure 2:**
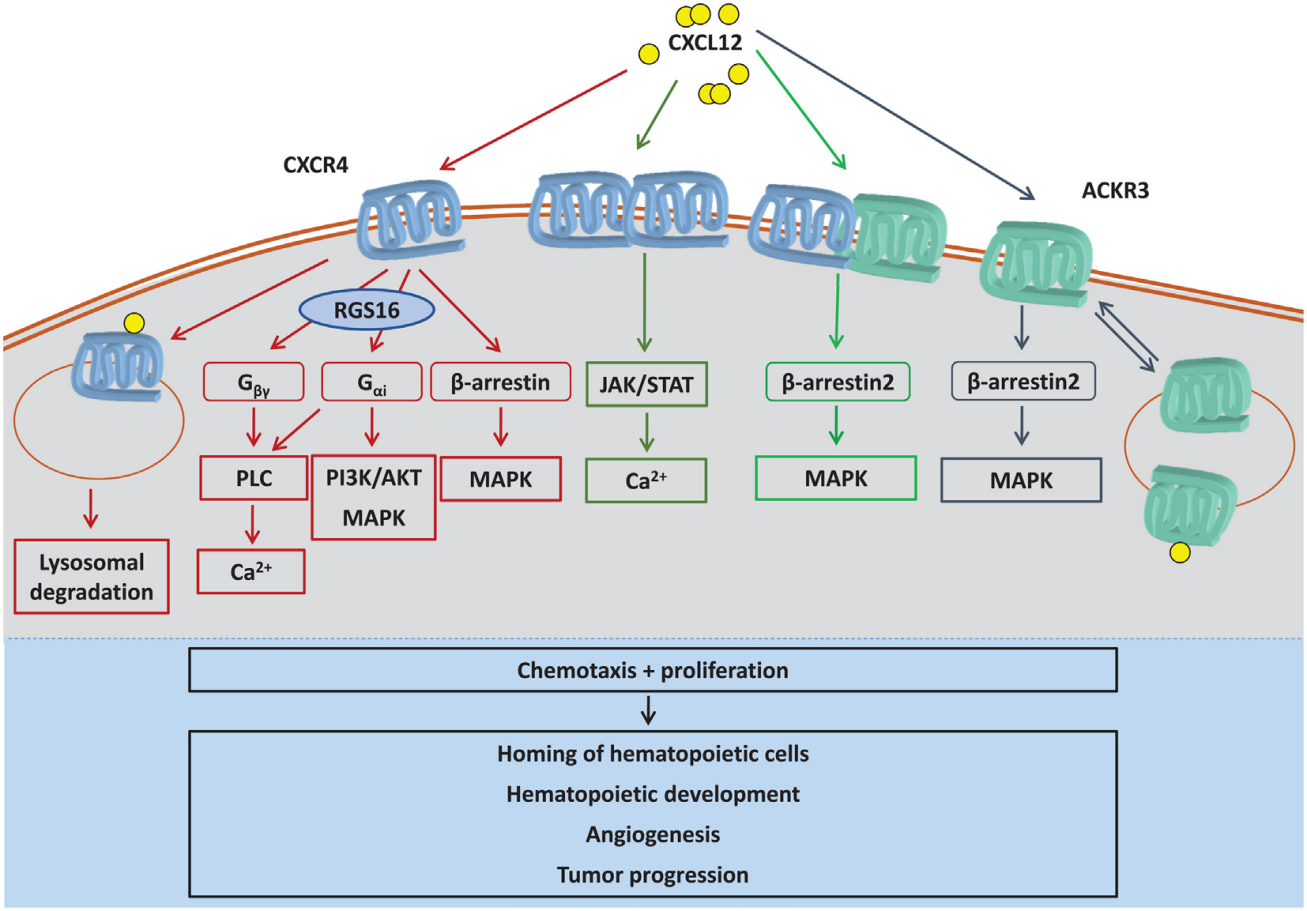
**CXCL12-induced signaling pathways**. CXCL12 can trigger intracellular signaling by binding to CXCR4 monomers, CXCR4 homodimers, ACKR3, or CXCR4/ACKR3 heterodimers. CXCR4 preferentially activates G protein-mediated signaling, which is negatively regulated by RGS16. The atypical chemokine receptor ACKR3 (previously called CXCR7) functions as a CXCL12 scavenger and also signals via β-arrestin. Also, complex formation between CXCR4 and ACKR3 shifts CXCL12-induced signaling away from classical G protein signaling to β-arrestin signaling. By CXCL12-induced dimerization, CXCR4 has also been reported to induce JAK/STAT signaling. Whereas CXCR4 is mostly degraded after CXCL12-elicited internalization, ACKR3 is continuously internalized and recycled to plasma membrane independent of ligand binding, a process that also promotes CXCL12 degradation. CXCL12 is known to induce chemotaxis and proliferation, supporting several downstream biological processes such as hematopoietic development, angiogenesis, or tumor progression. AKT, protein kinase B; Ca^2+^, calcium ions; G_α_, G protein subunit α; G_βγ_, G protein subunit βγ; JAK, janus kinase; MAPK, mitogen-activated protein kinase; PI3K, phosphatidylinositide 3-kinase; PLC, phospholipase C; RGS16, regulator of G protein signaling 16; STAT, signal transducer and activator of transcription.

CXCR4 is classified as a GPCR, indicating that one of the main pathways triggered by CXCR4 stimulation involves G protein-coupled signaling (Figure 2). The G protein complex is a heterotrimeric complex, composed of a Gα, Gβ, and Gγ subunit (Box 4), and is associated with CXCR4 and the inner leaflet of the plasma membrane. CXCR4 is mainly coupled to the Gα_i_ subunit, which after dissociation of the Gαβγ complex upon CXCR4 stimulation, inhibits adenylyl cyclase activity, and triggers mitogen-activated protein kinases (MAPK) and phosphatidylinositol-3-kinase (PI3K) pathway activation (96). The Gβγ subunit leads to the hydrolysis of phosphatidylinositol 4,5-bisphosphate (PIP_2_) to diacylglycerol (DAG) and inositol triphosphate (IP_3_) by phospholipase C (PLC) and subsequent mobilization of Ca^2+^ ions from intracellular stores (103, 104). This could also be considered a downstream effect of Gα_i_ activity, since the inhibition of Gα_i_ activity by its potent inhibitor pertussis toxin has been reported to lead to decreased Ca^2+^ mobilization from intracellular stores (103, 105). Although CXCR4 is most likely primarily coupled to Gα_i_, recent reports suggest that CXCR4 associates with other Gα subunits, i.e., Gα_q_ or Gα_12_, each of which has been associated with different intracellular signaling cascades (Box 4) (106).

Box 4 **G protein-coupled (GPCR) signaling.**

G protein-coupled signaling is a major signaling pathway in most cell types. The heterotrimeric G protein complex can interact with so-called GPCRs and is composed of the three subunits Gα, Gβ, and Gγ. In its non-active form, nucleotide guanosine diphosphate (GDP) is bound to the G protein complex. Upon ligand binding and subsequent activation and conformational change of the GPCR (107), GDP is replaced by guanosine triphosphate (GTP) and the three G protein subunits dissociate into a GTP-bound Gα monomer and a Gβγ dimer (108), each triggering distinct intracellular signaling pathways. After G protein activation, GTP is again hydrolyzed to GDP through an intrinsic GTPase activity of the Gα subunit, which in turn leads to re-association of the Gαβγ-trimer/GPCR complex and hence termination of GPCR signaling (96, 109). The G protein subunits can be divided into several classes, with more than 16 different Gα isoforms, 5 Gβ isoforms, and 14 Gγ isoforms [reviewed in Ref. (110)]. The Gβγ subunit leads to the hydrolysis of phosphatidylinositol 4,5-bisphosphate (PIP_2_) to diacylglycerol (DAG) and inositol triphosphate (IP_3_) by phospholipase C (PLC) and subsequent mobilization of Ca^2+^ ions from intracellular stores (103, 104). The Gα proteins are the main signal transducers, and association of GPCRs with different G_α_ isoforms leads to activation of different downstream signaling mediators. For example, Gα_s_ induces cyclic AMP (cAMP) production, activating protein kinase A (PKA) and thus the transcription factor cAMP-responsive element-binding protein (CREB). Both Gα_i_ and Gα_q_ are involved in activating PLC and subsequent mobilization of Ca^2+^ ions from intracellular stores, as well as inhibiting adenylyl cyclase activity and triggering activation of MAP kinases, the phosphatidylinositol-3-kinase (PI3K) pathway, and the NF-κB pathway (106).

### RGS16 as a negative regulator of CXCL12/CXCR4 signaling

G protein-coupled signaling triggers diverse downstream signaling pathways and hence needs to be tightly regulated. Signaling termination is initiated by hydrolysis of Gα-bound GTP to GDP by an intrinsic GTPase activity of the Gα subunit, with subsequent re-association of the inactive G protein trimer/receptor complex (96, 109). This GTPase activity can be further enhanced by so-called regulators of G protein signaling (RGS), which bind to the activated Gα subunit following GPCR stimulation. The family of RGS proteins consists of at least 37 members that all share a conserved 120 aa GTPase-accelerating domain (111), termed the RGS box (109). The family member RGS16 was identified as a negative regulator of CXCL12–CXCR4 signaling in 2005 (112). Overexpression of RGS16 leads to reduced CXCL12-induced migration of megakaryocytes and reduced activation of MAPK and the kinase AKT, whereas knockdown of RGS16 increased CXCR4 signaling (112). Furthermore, down-regulation of RGS16 expression by microRNA126 in endothelial cells was shown to increase CXCR4 signaling, leading to an auto-regulatory positive feedback loop through increased production of CXCL12 (113).

### Involvement of the JAK/STAT pathway in CXCL12/CXCR4 signaling

Upon binding of CXCL12, CXCR4 can dimerize and becomes phosphorylated at intracellular tyrosine residues by rapid recruitment and activation of the Janus kinases JAK2 and JAK3. These phosphorylated tyrosines mediate the binding of STAT proteins, which are phosphorylated by the CXCR4-bound JAK kinases, leading to STAT dimerization and initiation of the STAT signaling pathway (114) (Figure 2). The phosphorylation of CXCR4 by JAK2/3, and hence activation of the JAK/STAT pathway, is unaffected by treatment with the Gα_i_-specific inhibitor pertussis toxin. However, it is interesting to note that the complex of CXCR4 with JAK2/3 does not dissociate in pertussis toxin-treated cells, as it would normally do. This implies that the Gα_i_ protein could be involved in the recycling of the JAK/STAT receptor complex by uncoupling of JAK2/3 from CXCR4 (114). The precise mechanism for this effect however remains unidentified.

The activation of the JAK/STAT pathway leads to diverse cellular effects, including Ca^2+^ mobilization from intracellular stores, which shows again the complex interplay with G protein-coupled signaling. In fact, employing a JAK-specific inhibitor, it could be shown that the association of Gα_i_ to CXCR4 is dependent on JAK, further supporting a co-dependent mechanism of action between members of the JAK/STAT pathway and G protein-coupled signaling (115). Although activation of the JAK/STAT pathway by CXCR4 has interesting implications for future research, only limited data on this interaction are available to date.

### Beta-arrestin modulates CXCL12/CXCR4 signaling

Apart from classical signaling through G protein activation, CXCR4 has also been shown to induce β-arrestin-mediated signaling (Figure 2). β-Arrestin exists in two isoforms, β-arrestin-1 and β-arrestin-2, which have historically been described as terminators of G protein-coupled signaling. Signaling is terminated by recruitment of β-arrestin to the receptor site, whereby G protein coupling to the receptor is sterically hindered (116). In addition, β-arrestins facilitate the internalization of the receptor by acting as an adaptor for β(2)-adaptin and clathrin, transporting the receptor to clathrin-coated pits for endocytosis (117). Interestingly, high levels of intracellular CXCR4, located in early and recycling endosomes, can be found constitutively in HSPCs, independent of ligand binding. This suggests that constitutive endocytosis of CXCR4 and possibly CXCR4 recycling to the cell membrane, mediated by clathrin-coated vesicles, are important mechanisms in HSPC regulation and trafficking (118). Similar findings were reported in fetal mesenchymal stem/stromal cells (119).

However, β-arrestin is not only involved in G protein-coupled signaling termination and GPCR internalization, but also initiates signaling itself. One of the first findings pointing to signaling mediated by β-arrestin was that β-arrestin acts as an adapter protein between SRC family tyrosine kinases and a GPCR, which in turn leads to activation of the MAP kinases ERK 1/2 (120). Since then, a variety of β-arrestin-mediated signaling effects have been described, including scaffolding for AKT, PI3K, and phosphodiesterase 4 (PDE4) in the context of specific receptors [reviewed in Ref. (116)]. G protein-independent activation of ERK 1/2 through the β adrenergic receptor (β2AR) was reported in 2006 by Shenoy et al. (121). Treatment of HEK293 cells with isoproterenol, which activates β2AR signaling, led to distinct ERK1/2 activation outcomes: rapid ERK1/2 activation sensitive to pertussis toxin treatment and therefore Gα_i_ protein-dependent, as well as a slower, more sustained ERK1/2 activation which was insensitive to pertussis toxin and thus Gα_i_ protein-independent, and which could be abrogated by siRNAs against β-arrestins (121). These findings show that the involvement of β-arrestin-mediated signaling in the context of GPCRs should not be underestimated. When it comes to the function of β-arrestin in regard to CXCL12, it clearly emerges that this aspect cannot be investigated without taking the interaction of CXCR4 and ACKR3 into account (122–124), as discussed in more detail below.

### Homo-oligomerization of CXCR4 and CXCL12

An important event in the activation of GPCRs is receptor dimerization, a process that is of great interest for drug targeting (125). Crystallization of CXCR4 in complex with the antagonists IT1t and CVX15 or the viral protein ligand vMIP-II revealed the existence of a CXCR4 dimer (11, 24). Also, stable, constitutive dimerization and even higher-order oligomerization of CXCR4 in the absence of a ligand could be shown in fluorescence resonance energy transfer (FRET) experiments (126, 127), and binding of the ligand CXCL12 was reported to even further increase CXCR4 oligomerization in FRET experiments (128).

Not only CXCR4, but also CXCL12 can form homodimers, and crystallization studies of CXCR4 left open the possibilities of CXCL12 interacting as monomer as well as dimer with a CXCR4 dimer (24). Of note, the oligomerization state of CXCL12 has been reported to influence signaling, however with conflicting data presented. Ray et al. report on a bias for monomeric CXCL12 to initiate G protein-coupled signaling and for dimeric CXCL12 to rather promote recruitment of β-arrestin-2 to CXCR4, as shown *in vitro* in breast cancer cells (129). By contrast, Drury et al. showed in colon carcinoma cells that dimeric CXCL12 has merely any effect on β-arrestin, whereas monomeric CXCL12 recruited β-arrestin. In this study, both CXCL12 forms however mobilized intracellular calcium and inhibited adenylyl cyclase signaling, showing activation of Gα_i_ signaling (130). These findings show that a clear-cut distinction of the dimerization state of CXCL12 and its relation to specific downstream signaling pathways cannot (yet) be made. Furthermore, to which extent dimerization of CXCL12 is favored over the monomeric form *in vivo* still remains open and is believed to be highly tissue-dependent (129).

### Interaction of CXCR4 and CXCL12 with other signaling mediators

Apart from homodimerization (24, 126, 127), CXCR4 forms a functional MIF receptor complex with CD74 (131) and can form heterodimers with the chemokine receptor ACKR3 (previously CXCR7), modulating CXCL12/CXCR4 signaling as will be discussed in detail below. Furthermore, oligomerization of CXCR4 with CCR5 and CCR2, chemokine receptors important for HIV infection, has been shown by FRET and co-immunoprecipitation experiments (132–137).

Also, it could be shown that CXCR4 forms a heterodimer with the T-cell receptor (TCR) upon stimulation of T-cells with CXCL12, leading to several cellular outcomes such as ERK activation, increased Ca^2+^ levels, gene transcription, and cytokine production (138). For example, a particular factor regulated by CXCL12 signaling in a TCR-dependent way is RasGRP1, a Ras guanine-nucleotide exchange factor, which activates ERK and has important functions in autoimmunity and immunodeficiency (139). Interestingly, the heterodimerization of TCR and CXCR4 was found to be crucial for CXCR4 endocytosis in T-cells (140).

Furthermore, in an intriguing study conducted by Wagner et al., it was shown that in a mouse model of hindlimb ischemia, the administration of a TLR2 blocking antibody had similar pro-angiogenic effects compared with administration of CXCL12 and induced the activation of both ERK1/2 and AKT, the canonical signal transduction pathways of CXCR4. Immunoprecipitation experiments revealed an interaction of TLR2 and CXCR4 in endothelial cells and it was proposed that the effects of the TLR2 blocking antibody were mediated through CXCR4. Indeed, upon CXCR4 knockdown or G protein inhibition, the observed pro-angiogenic effects of the TLR2 blocking antibody were abolished, supporting a role for a functional interaction of CXCR4 with TLR2 in mediating pro-angiogenic effects of TLR2 blocking (141).

Furthermore, recent research implicates a functional interaction of a CXCR4/ACKR3 dimer with the androgen receptor, regulating CXCL12-dependent cellular motility in a prostate tumor cell line (142). Also, heteromerization of CXCR4 with α1A/B-adrenergic GPCR has been reported recently in HeLa cells and human vascular smooth muscle cells (143). In addition, interactions of CXCR4 with CD4, lipopolysaccharide receptor, Epstein–Barr virus-encoded GPCR BILF1, kappa-type opioid receptor, and delta-type opioid receptor have been described [reviewed in Ref. (144)].

Finally, not only CXCR4 but also CXCL12 has been shown to interact with other signaling mediators affecting CXCL12/CXCR4 signaling: high mobility group box 1 (HMGB1), a damage-associated molecular pattern released from damaged cells, was revealed to form a heterocomplex with CXCL12, shifting the efficiency of CXCR4 activation to lower concentrations and mediating inflammatory cell recruitment *in vivo* (145).

Together, these findings show that the CXCL12/CXCR4 axis is not monogamous, but is connected to a variety of other signaling receptors.

## Biological Processes and Signaling Triggered by the MIF/CXCR4 Axis

Migration inhibitory factor is involved in the progression of diverse acute and chronic inflammatory diseases including septic shock, atherosclerosis, septic lupus erythematosus, bladder pain, and allergic diseases such as eosinophilic esophagitis (19, 146–149). On the other hand, MIF also exhibits protective functions, e.g., in liver fibrosis and myocardial ischemia-reperfusion injury, associated with the activation of the protective kinases AMPK and PKCε (150–153). Recently, a novel protective role of MIF was revealed in the developing cerebral cortex upon tissue damage, with MIF upregulated upon cell death and stimulating the proliferation of microglia in the developing cerebral cortex (154).

**Figure 3:**
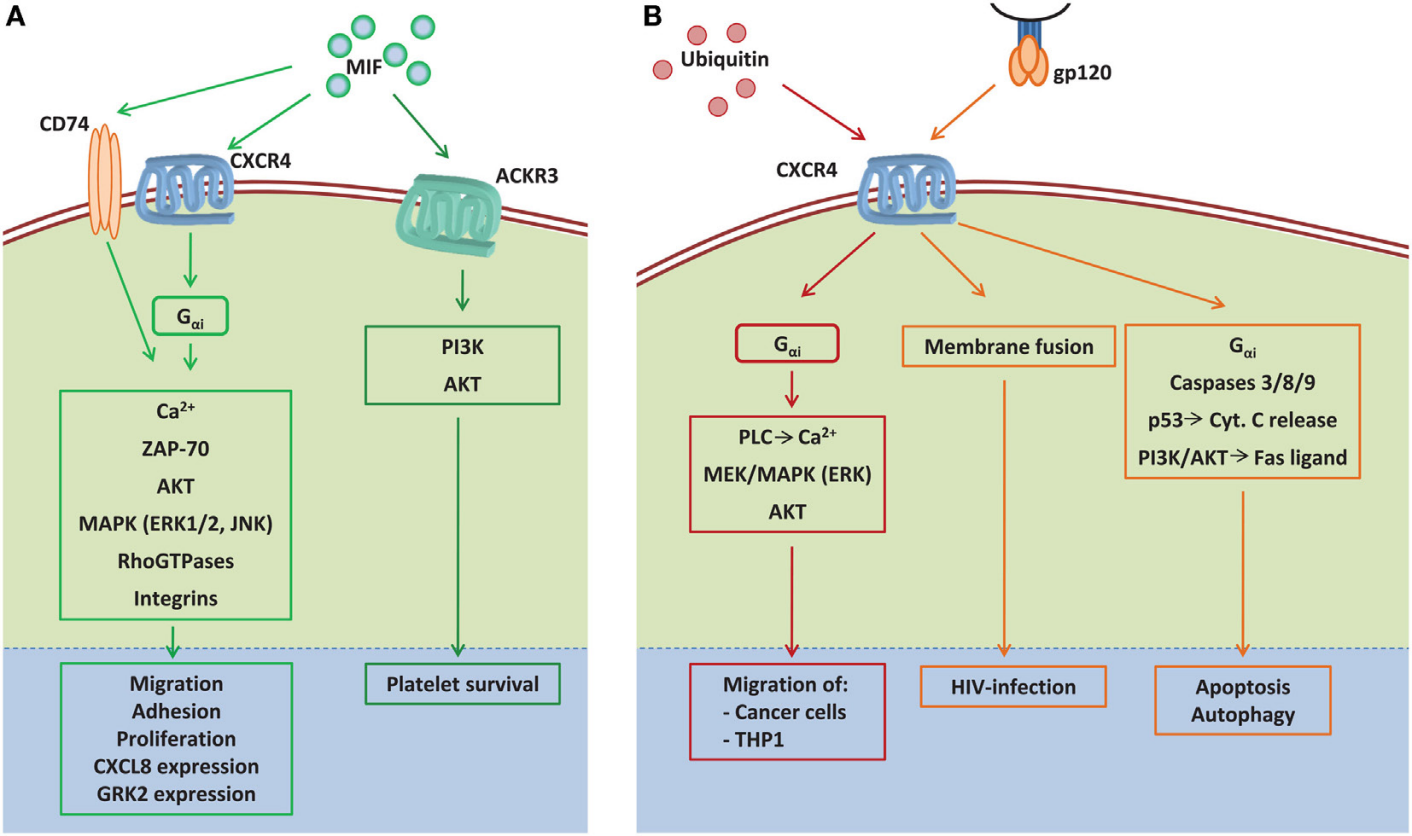
**The signaling network induced by the non-canonical CXCR4 ligands MIF, extracellular ubiquitin, and gp120**. **(A)** MIF induces signal transduction by binding to the CXCR4, which can form a receptor complex with CD74 under certain conditions, or by binding to the atypical chemokine receptor 3 (ACKR3) (previously called CXCR7). MIF binding to CXCR4 triggers cytosolic Ca^2+^ influx, integrin activation, and ZAP-70 activation via G_αi_, resulting in cell adhesion, migration, proliferation, and GRK2 expression. By binding to the CXCR4/CD74 signaling complex, MIF activates ERK1/2 and AKT signaling promoting cell survival and proliferation. Additionally, the MIF–CXCR4/CD74 interaction promotes JNK phosphorylation, which results in enhanced CXCL8 expression. By binding to ACKR3 MIF induces platelet survival via the activation of PI3K and AKT. **(B)** By binding to CXCR4, ubiquitin induces the migration of cancer as well as THP1 cells through the activation of G_αi_-dependent PLC-induced Ca^2+^ influx, MAPK, and AKT phosphorylation. The HIV glycoprotein gp120 uses CXCR4 as co-receptor for fusion of viral and host cell membrane, which results in HIV infection. In addition, gp120 can induce cell death via CXCR4 by activating caspases, p53-dependent cytochrome C release, and PI3K/AKT-dependent Fas ligand expression. AKT, protein kinase B; Ca^2+^, calcium ions; Cyt. C, cytochrome C; ERK1/2, extracellular signal-regulated kinases 1/2; G_αi_, G_i_-protein subunit α; gp120, glycoprotein 120; GRK2, G protein-coupled receptor kinase 2; JAK, janus kinase; MAPK, mitogen-activated protein kinase; MEK, mitogen-activated protein kinase kinase; MIF, macrophage migration inhibitory factor; PI3K, phosphatidylinositide 3-kinase; PLC, phospholipase C; THP1, monocytic cell line; ZAP-70, zeta-chain-associated protein kinase 70.

*CD74*, the surface-expressed form of MHC class-II-associated invariant chain, has been identified as a MIF receptor. In a signaling complex with CD44, MIF/CD74 interaction leads to ERK1/2 and AKT phosphorylation promoting cell survival and proliferation (155–157) (Figure 3A). The chemotactic properties of MIF are mediated via binding to the chemokine receptors CXCR2 and CXCR4 (4), in part by interaction with CD74. Interaction of MIF with *CXCR2* has been shown to mediate the recruitment and arrest of monocytes and neutrophils (4, 158), with MIF–CXCR2-induced signaling discussed in detail previously (19). Via *CXCR4*, MIF has been shown to recruit many cell types, including T-cells, B-cells, eosinophils, endothelial progenitor cells, mesenchymal stromal cells, as well as cancer cells (4, 148, 159–162). Furthermore, MIF–CXCR4 interaction increases in experimental bladder inflammation (163), with PAR4-induced abdominal hypersensitivity shown to occur through MIF and at least partially also through CXCR4 on the urothelium (149). However, the underlying signaling events of MIF–CXCR4 association are only partially enlightened.

In T- and B-cells, MIF-induced cell migration is G_iα_-dependent and MIF induces cytosolic Ca^2+^-influx (4, 161). Furthermore, MIF triggers the rapid activation of integrins, as shown in T-cells (4), and which induces cell arrest by binding to integrin ligands presented by, e.g., activated endothelium. In B-cells, both MIF- and CXCL12-induced migration are dependent on the participation of the tyrosine kinase ZAP-70 (161). ZAP-70 was originally noted for its involvement in the initiation of the antigen-dependent T-cell response by phosphorylating the scaffold proteins LAT and SLP-76 (164). During T-cell migration triggered by CXCL12, ZAP-70 regulates directionality by interacting with talin, which acts as an integrin scaffold for F-actin (165). Whether ZAP-70 exerts these same functions in MIF-induced signaling remains to be unraveled.

In colon cancer cells, the MIF/CXCR4 axis induces an aggressive phenotype by inducing proliferation, adhesion, migration, and invasion of these cells, which is related to metastasis (160, 166). Similarly, the MIF/CXCR4 axis was recently shown to be the main axis mediating the recruitment of mesenchymal stromal cells to tumors and underlying their invasion capacity, both *in vitro* as well as in a pulmonary metastasis model, and this was linked with signaling through the MAP kinases MEK1/MEK2, upstream of ERK1/2 (162). On the other hand, CXCR4-expressing human rhapdomyosarcoma cells do not only show phosphorylation of ERK1/2 and AKT, but also enhanced cell adhesion after stimulation with MIF, the latter preventing their release into the circulation and thereby inhibits metastasis (7). Therefore, the role of MIF in metastasis seems to be ambivalent maybe dependent on tumor type. Interestingly, MIF, which is secreted in response to hypoxia by different tumor cells, was reported to induce CD11b^+^GR1^+^ myeloid cell migration via CD74/CXCR4 and CD74/CXCR2 complexes, as well as through p38 and PI3K activation (167). Because CD11b^+^GR1^+^ myeloid cells have gained much attention for their role in tumor immunity suppression as well as for their ability to promote angiogenesis (168, 169), MIF could support tumor growth by recruiting CD11b^+^GR1^+^ myeloid cells.

In addition, tumor-originated MIF leads to enhanced accumulation of interleukin-17-producing subsets of tumor-infiltrating lymphocytes by binding to CXCR4, which can affect patient prognosis (170). The clinical relevance of these interleukin-17-producing lymphocyte subsets for cancer seems to be cancer type-dependent. While these subsets are beneficial in ovarian cancer, the number of interleukin-17-producing subsets negatively predicts the outcome of patients with hepatocellular carcinoma (171, 172).

In 2009, Schwartz et al. could show that CXCR4 and CD74 form a functional MIF receptor complex, which induces MIF-dependent AKT phosphorylation that can be blocked by anti-CXCR4 as well as by anti-CD74 antibodies (131) (Figure 3A). After binding to this complex, MIF is internalized by clathrin/dynamin-dependent endocytosis leading to endosomal AKT signaling (173). This CXCR4/CD74-complex also mediates MIF-dependent JNK activation via the PI3 and SRC kinases leading to c-Jun and AP-1 activation to upregulate gene expression of CXCL8 in T-cells (174).

Despite its chemotactic activity, MIF was originally described as a substance which inhibits the random movement as well as directed migration of macrophages, hence its name “macrophage migration inhibiting factor” (175, 176). Later on, this was explained by a desensitization effect of MIF toward migration induced by other chemokines, as also shown, e.g., MCP1/CCL2- and CXCL8-induced migration (4, 177). Recently, it was suggested that MIF inhibits migration of human monocytic U937 cells through CXCR4 in the absence of CD74 via a perturbation of RHO GTPase signaling. MIF/CXCR4 interaction was shown to induce G protein-coupled activation of RHO-A followed by subsequent inactivation. In addition, RAC1 was transiently inactivated while Cdc42 showed cyclic activation and inactivation. Together, these results were suggested to contribute to MIF-induced migration inhibition in U937 cells. Furthermore, they indicate that CXCR4 can mediate MIF signaling in the absence of CD74, in addition to its function as MIF co-receptor in complex with CD74 (178).

Finally, increased levels of MIF, as observed in plasma of diabetic patients, also enhance the expression of GRK2 in cardiomyoblasts, an effect mediated through CXCR4 (179). As an upregulation of GRK2 in cardiomyocytes precedes the development of heart failure (180), this finding might suggest that MIF could promote heart failure by amplifying GRK2 expression.

## CXCR4 Signaling Triggered by the Non-Canonical Ligands eUb and gp120

### Extracellular ubiquitin (eUb)/CXCR4 signaling

Opposite to the well-characterized functions of ubiquitin as intracellular signaling molecule, as discussed earlier, only little attention has been paid to its extracellular actions. eUb can be found in human serum in concentrations varying in response to different diseases. The cellular source and the underlying release mechanism remain unclear (181, 182), but active secretion of ubiquitin was assumed after increased ubiquitin concentrations were measured in the supernatant of Ba/F3 B-cells and 293T human embryonic kidney cells after transfection with an ubiquitin expression vector (183). Furthermore, it has been shown that eUb can be taken up by cells (184), and induces Ca^2+^ flux after binding to THP1 monocytes. Ubiquitin-induced Ca^2+^ flux could be inhibited by the use of the G protein inhibitor pertussis toxin, indicating that ubiquitin signals via a GPCR. By gene silencing and the use of the CXCR4 antagonist AMD3100 Saini et al. showed that eUb signals via CXCR4 and leads to Ca^2+^ flux and reduced cAMP levels (70). Ubiquitin-induced Ca^2+^ flux can also be attenuated by the PLC inhibitor U73122, indicating that PLC plays a role in ubiquitin-induced signaling events (185) (Figure 3B).

Extracellular ubiquitin has chemotactic properties inducing the migration of different cancer cell types as well as THP1 cells via CXCR4, PLC, AKT, and the MEK/ERK pathway (186–188). The effects of ubiquitin and the canonical CXCR4 ligand CXCL12 are partially synergistic: co-stimulation with suboptimal ligand concentrations leads to enhanced Ca^2+^ flux, without synergistic effects on cAMP levels, AKT and ERK1/2 phosphorylation or chemotactic responses (188). In 2011, ubiquitin has been characterized as a substrate for the ubiquitously expressed cell surface protein “insulin degrading enzyme” (IDE), cleaving the C-terminal di-Gly aa from ubiquitin (189). This modification modulates CXCR4-mediated ubiquitin signaling. Reduced IDE expression enhances the ubiquitin-induced reduction in cAMP levels and reinforces the chemotactic activity of ubiquitin (186). Modulation of ubiquitin by IDE could thereby contribute to the fine-tuning of CXCR4-mediated cell functions.

### Gp120/CXCR4 signaling

Binding of gp120 on the surface of the HI-virus to CD4 and the HIV co-receptors CCR5 or CXCR4 leads to the fusion of the viral and the host cell membrane (79). Also, binding of gp120 to CXCR4 is involved in HIV-mediated apoptosis of infected and uninfected lymphocytes (190), neurons (191), cardiomyocytes (192), hepatocytes (193), and different cancer cells (194, 195). The underlying signaling events are controversially discussed (Figure 3B). CXCR4 signaling is typically mediated via the Gα_i_ protein. But in case of gp120/CXCR4-mediated apoptosis, the signaling pathway seems to be Gα_i_ protein-independent, at least in T-cells (196, 197), whereas gp120/CXCR4-induced apoptosis of breast cancer cells (194) and hepatocytes (193) depends on Gα_i_ protein involvement. Also, the participation of caspases seems to be cell type-dependent: caspases 8 and 9 play an essential role in gp120/CXCR4-induced apoptosis of cardiomyocytes (192); gp120/CXCR4-induced apoptosis of hepatocytes is caspase-independent (193); but T-cells seem to display a caspase-independent pathway as well as a pathway involving caspases 3 and 9 (197, 198). By the use of CD4 inhibitors or CD4 mutants, different studies reported that gp120/CXCR4-induced apoptosis is CD4-independent. By contrast, CD45 was observed to be important for gp120/CXCR4-mediated T-cell apoptosis, and through interaction with CXCR4, CD45 is involved in Fas ligand activation via the PI3K/AKT pathway (199). Gp120 expression on the surface of infected cells can also cause apoptosis by binding to a CD4/CXCR4-expressing cell. The resulting “fused cell,” also called syncytium, undergoes apoptosis mediated by mitochondrial membrane permeabilization and cytochrome c release involving p53 activation (200, 201).

To gain more insight into the molecular mechanisms leading to gp120/CXCR4-mediated apoptosis of lymphocytes, Molina et al. performed a proteomic analysis of immune cells after coculture with cells expressing gp120. This approach showed that most proteins involved in gp120/CXCR4-mediated apoptosis can be linked to degradation processes, redox homeostasis, metabolism, or cytoskeleton dynamics (202). Not only apoptosis but also autophagy of CD4 T-cells can be induced by gp120 binding to CXCR4, but the underlying signaling events remain unclear (203). Because gp120/CXCR4-induced lymphocyte death leads to pathological immunodeficiency, a lot of research is still ongoing to identify a CXCR4 antagonist that would selectively prevent the gp120/CXCR4 interaction and thereby HIV-triggered disease progression (204).

## ACKR3 Influences Signaling through CXCR4

Atypical chemokine receptor 3 (ACKR3) (originally named RDC-1 or CXCR7) was first described in 1989 as a putative GPCR cloned from thyroid complementary DNA (205). Further evidence for RDC-1 belonging to the seven-transmembrane-spanning receptor family was obtained by sequence analysis (206). The notion that RDC-1 might be part of the CXC family of chemokine receptors was brought up by Heesen et al., who reported in 1998 a 55% nucleic acid similarity between mouse RDC-1 and rabbit CXCR2 (207). In 2005, Balabanian et al. identified the orphan receptor RDC-1 as another receptor for CXCL12 and hence suggested to rename it to CXCR7 according to the chemokine nomenclature (6). In 2014, the name was changed into ACKR3 (12). Of note, ACKR3 was reported to have a ~10-fold higher affinity for CXCL12 compared with CXCR4 (6, 36, 208). This finding has considerably changed the view of CXCL12/CXCR4 signaling, as CXCR4 was until then believed to be the only receptor for CXCL12, and since, ACKR3 has been under intensive investigation.

ACKR3 is expressed in a variety of tissues, including, for example, embryonic neuronal and heart tissue, hematopoietic cells, and activated endothelium (209). Its expression has also been linked to a variety of cancers, including, for example, breast and lung tumors (210), brain metastases (211), and renal cell carcinoma (212). Apart from cancer research, ACKR3 has been implicated in other diseases, for example in acute coronary syndrome, in which platelets from patients showed enhanced surface expression of ACKR3 but not CXCR4 (213).

### CXCL12 as a ligand for ACKR3 and interrelation with CXCL12/CXCR4 signaling

With ACKR3 as a high-affinity receptor for CXCL12 (6, 36, 208), ACKR3 functions as a scavenger receptor for CXCL12, thereby downtuning classical CXCL12/CXCR4 signaling. On the other hand, ACKR3 is also able to induce CXCL12 signaling independently from CXCR4 (6, 214–216), as will be discussed in more detail below. It is currently thought that ACKR3 does not *per se* induce G protein signaling upon binding of CXCL12, based on which the previous name CXCR7 was recently renamed into ACKR3 based on its classification in the group of ACKRs that do not signal through G proteins (12), although this has been challenged by others (217).

ACKR3 continuously cycles between the plasma membrane and intracellular compartments without or with ligand binding, and in contrast to CXCR4, ACKR3 is not degraded after internalization (209) (Figure 2). ACKR3 internalization is dependent on β-arrestin and also involves constitutive ubiquitination for correct trafficking of ACKR3 (218).

Co-expression of CXCR4 with ACKR3 resulted in heterodimerization independent of ligand binding (219). This was associated with a constitutive recruitment of β-arrestin-2 to the CXCR4/ACKR3 complex, with simultaneous down-regulation of Gα_i_-mediated signaling as shown by a cAMP reporter gene assay as read-out of Gα_i_ signaling (123) (Box 4; Figure 2). Based on these results, Decaillot et al. postulated that the CXCR4/ACKR3 heterodimer down-tunes classical CXCL12/CXCR4-triggered G protein-coupled signaling by preferentially triggering the recruitment of β-arrestin and hence inducing β-arrestin-mediated signaling pathways, including activation of the MAP kinases ERK1/2, p38 and SAPK. The activation of these pathways is enhanced by binding of CXCL12 to the receptor complex, which leads to an increased recruitment of β-arrestin compared to the interaction of CXCL12 with CXCR4 alone (123). Of note, CXCR4/ACKR3 heterodimers form as efficiently as CXCR4 homodimers, indicating that these mechanisms are of equal importance (219).

Furthermore, recent reports showed additional negative regulatory functions of ACKR3 toward classical CXCL12/CXCR4 signaling: an agonist of ACKR3 promoted the dimerization of ACKR3 with CXCR4, which in turn led to internalization and degradation of CXCR4 and inhibition of CXCL12-induced tube formation (124). Also, ACKR3 binding to CXCL12 but also to its other ligand CXCL11 (as discussed below) enhances internalization of the chemokine ligand/receptor complex and results in lysosomal degradation of the chemokine ligand (209, 220), which again negatively affects CXCL12/CXCR4 signaling. Together, these findings have led to the current idea that the CXCR4/ACKR3 complex results in decreased classical signaling of CXCL12/CXCR4 through G protein-coupled pathways and initiation of β-arrestin signaling through ACKR3.

Of note, ACKR3 preferentially sequesters the monomeric form of CXCL12, whereas dimeric CXCL12 showed significantly lower binding to ACKR3 *in vitro* and in a breast cancer xenograft model (129). However, how the oligomerization state of CXCL12 influences downstream signaling remains controversial, as discussed earlier. Taking into account the findings by Drury et al. who showed in colon carcinoma cells that monomeric but not dimeric CXCL12 preferentially recruited β-arrestin to CXCR4 (130), one could hypothesize that enhanced β-arrestin signaling through ACKR3 is due to preferential binding of monomeric CXCL12. However Ray et al. reported that monomeric CXCL12 actually promotes Gα_i_ signaling through CXCR4, whereas dimeric CXCL12 rather recruited β-arrestin (129). This shows that these mechanisms still need to be further investigated and could possibly be cell type-dependent and influenced by the build-up of different receptor complexes, as, for example, homomeric or heteromeric interactions involving CXCR4, ACKR3, and even CD74.

Furthermore, different reports come to the conclusion that CXCL12 can signal through ACKR3 independently of CXCR4, further diversifying the potential mechanisms of the CXCL12 signaling network (Figure 2). The migration of neural progenitor cells was shown to be regulated by CXCL12, both through the well-established mechanism of CXCR4 but also independently of CXCR4 via ACKR3 (216). Similarly, CXCL12 can induce T-cell migration through ACKR3 independent of CXCR4 (6, 215), and in cortical interneurons MAPK activation can be induced by CXCL12/ACKR3 independent of CXCR4 (214). However, the idea that ACKR3 is merely a scavenger receptor modulating CXCR4 function and an ACKR not coupled to classical G protein signaling was in turn challenged by Ödemis et al., who showed that ACKR3-mediated effects on ERK and AKT activation in rodent astrocytes and human glioma cells were pertussis toxin-sensitive, and hence mediated by G protein activation (217).

In conclusion, the previous view of classical CXCL12/CXCR4 signaling needs to take into account active signaling moderation by ACKR3. Whether CXCL12 signaling is mediated through CXCR4, ACKR3, or both receptors in conjunction seems to be cell type-dependent (221). Interestingly, a recent report described CXCL12 to stimulate CXCR4 internalization on platelets, which in turn led to increased ACKR3 surface expression. This CXCR4-dependent increased ACKR3 surface exposure had anti-apoptotic effects on platelets (222). These findings reveal a highly interesting, novel pro-survival mechanism of CXCL12, and also demonstrate that CXCR4 and ACKR3 can work in close conjunction to trigger specific biological effects. However, signaling through CXCR4 vs. ACKR3 can also have opposite biological effects. For example, it was recently shown that ACKR3 is upregulated in liver sinusoidal endothelial cells after acute liver injury and in cooperation with CXCR4 induces liver regeneration through the production of pro-regenerative angiocrine factors. By contrast, chronic liver injury increased CXCR4 expression, overwhelming ACKR3 signaling and promoting a pro-fibrotic response instead (223).

### CXCL11 as a ligand for ACKR3

In 2006, ACKR3 (at that time still called CXCR7) was also identified as a receptor for the chemokine CXCL11, which was previously believed to exclusively bind to CXCR3 (208, 224). This chemokine was discovered in 1998 by Cole et al. by sequence analysis of cDNAs derived from cytokine-activated primary human astrocytes (224), and based on experiments named as interferon-inducible T-cell alpha chemoattractant (I-TAC). Later on, this name was replaced by CXCL11, according to the common chemokine nomenclature (225, 226). Interactions of CXCL11 with CXCR3 have been intensively investigated, and have, for example, been associated with several pro- as well as anti-tumorigenic effects (227). By contrast, the CXCL11/ACKR3 relationship is less well studied. Yet, it clearly emerges that CXCL12, CXCL11, and their common receptor ACKR3 form an interactive network. For example, local administration of CXCL11 in a mouse model of colorectal cancer enhanced tumor growth. Without exogenous CXCL11 stimulation, blocking endogenous CXCL11 or CXCL12 alone did not influence tumor growth and angiogenesis, whereas the combined inhibition almost completely abrogated tumor angiogenesis, providing evidence for the proposed close relationship of these chemokines (228).

### Potential alternative ligands for ACKR3: MIF and gp120

Apart from the binding of CXCL12 and CXCL11, ACKR3 has also been implied as a receptor for MIF (Figure 3A). For example, an anti-ACKR3 antibody inhibited the adhesion of rhabdomyosarcoma cells in response to MIF in an *in vitro* assay (7). Furthermore, a recent report indicated MIF to interact with both CXCR4 and ACKR3 on the platelet surface, although biochemical receptor binding evidence is currently still elusive. Although MIF could also induce CXCR4 internalization similar to CXCL12, MIF was not able to induce downstream ERK phosphorylation upon CXCR4 binding and also failed to induce subsequent upregulation of ACKR3 externalization, likely due to lack of CD74, which is not expressed in platelets. However, MIF-induced platelet survival through ACKR3 but not CXCR4, as well as activation of the PI3K–AKT pathway (229). The injection of MIF in a mouse model led to decreased thrombus formation after arterial injury, which could be abrogated by a ACKR3-blocking antibody. *In vitro*, reduced thrombus formation by MIF was mediated through both CXCR4 and ACKR3 (229).

Finally, the similarity of ACKR3 to CXCR4 in ligand binding is also apparent by the ability of both receptors to act as co-receptors for several strains of HIV in combination with CD4 (230). While for CXCR4, binding of the HIV envelope glycoprotein gp120 is a well-established mechanism for viral entry (231), interaction of HIV envelope proteins to ACKR3 has not yet been demonstrated by biochemical receptor binding assays.

## Involvement of (Defective) CXCR4 Signaling in Pathological Settings

Since CXCR4 signaling is induced by different ligands and affects important biological processes, it is not surprising that CXCR4 is also involved in a plethora of pathological events, such as HIV infection (15, 179), WHIM syndrome (232), as well as diverse cancer types (96).

As already introduced before, CXCR4 is important for HIV entry into T-cells of infected patients, and this conclusion is underlined by the fact that administration of the CXCR4 antagonist AMD3100/Plerixafor can stop virus replication (9). Therefore, CXCR4 antagonists are still under thorough investigation for their potential therapeutic value in HIV infection (233).

The warts, hypogammaglobulinemia, infection, and myelokathexis (WHIM) syndrome was first described in 1990 by Wetzler et al. (234). WHIM syndrome is characterized by severe neutropenia despite having abundant mature myeloid cells in the bone marrow, which is termed myelokathexis (232). Gain-of-function mutations in the *CXCR4* gene were identified as the underlying cause of this autosomal-dominant syndrome in 2003 (232, 235), corresponding to the fact that CXCR4 mediates neutrophil retention in the bone marrow (93). A highly interesting finding on WHIM was published this year; McDermott et al. report a case in which a patient with WHIM was spontaneously cured by chromothripsis, an intensive deletion and rearrangement process in chromosomes. Here, the defective *CXCR4* gene was randomly deleted in a hematopoietic stem cell that repopulated the myeloid but not the lymphoid lineage, leading to complete remission of the patient (235).

Furthermore, CXCR4 is the chemokine receptor most widely expressed in malignant tumors (236). It plays a role in a variety of cancer types, and has been linked with cancer cell proliferation and metastasis to bones and lymph nodes through both CXCL12 and MIF, as described above (96, 160, 166). For example, activation of CXCR4 induces leukemia cell trafficking and homing to the bone marrow (237) and is critical for the growth of both malignant neuronal and glial tumors (238).

In contrast to pathological effects of CXCR4 signaling, CXCR4 also exerts important protective functions in the context of disease. For example, the CXCL12/CXCR4 axis exerts cardioprotective effects after myocardial ischemia by enhancing the incorporation of progenitor cells in the infarcted region and promoting survival of cardiomyocytes (98, 99, 239). On the other hand, CXCR4 also promotes the recruitment of inflammatory cells to the infarcted heart (240, 241) and has in this context been linked to an increase in infarct area (240). This indicates a double-edged role of CXCR4 in myocardial ischemia (241), which has also been described for MIF (242), as discussed in detail in recent reviews (1, 243), and warns for a careful evaluation of effects of CXCR4 antagonists in clinical trials.

## Development and Clinical Use of CXCR4 Antagonists

Since CXCR4 signaling is involved in a plethora of pathological processes, small-molecule antagonists directed against CXCR4 are of great interest for medical treatment. A large variety of drug candidates and lead compounds targeting CXCR4 have been discovered over the last years, with several classes of chemical compounds being investigated. While peptide-derived compounds were the earliest anti-CXCR4 agents under investigation, they had poor pharmacokinetic properties. Nevertheless, these compounds were essential for the development of a basic pharmacophore model to further develop more intricate smaller molecules antagonizing CXCR4. These include cyclic pentapeptide-based antagonists, indole-based antagonists, tetrahydroquinolines-based antagonists, para-xylyl-enediamine-based compounds, guanidine-based antagonists, quinoline derivatives, and various other compounds, as reviewed in detail recently (2). The first and so far only CXCR4 antagonist that was approved by the Food and Drug Administration (FDA) is AMD3100, a bicyclam compound marketed under the brand name Plerixafor (Genzyme Corporation). It is being used in combination with G-CSF for the mobilization of HSPCs for autologous transplantation in patients with non-Hodgkin’s lymphoma (244). The use of HSPCs, derived by treatment with G-CSF and AMD3100, has essentially replaced bone marrow as a source of stem cells for both autologous and allogeneic transplantation, providing greater safety for the patient (245). In addition to the FDA-approved Plerixafor, other CXCR4 antagonists are being evaluated in ongoing clinical trials. To date, nine other clinical trials with CXCR4 antagonists are listed in the database at clinicaltrials.gov (search term “CXCR4”) (Table 1).

**Table 1:** **Ongoing clinical trials with CXCR4 antagonists as listed in the database at clinicaltrials.gov (search term “CXCR4”)**.

Drug	Clinical trial phase	Being tested in	Sponsor
BMS-936564	Phase 1	Multiple myeloma	Bristol–Myers Squibb
BKT140	Phase 1/phase 2	Multiple myeloma	Biokine Therapeutics Ltd
BL-8040	Phase 1/phase 2	Chronic myeloid leukemia	Sheba Medical Center
POL6326	Phase 2	Large reperfused ST-elevation myocardial infarction	Polyphor Ltd.
BMS-936564	Phase 1	Acute myelogenous leukemia; diffuse large B-cell leukemia; chronic lymphocytic leukemia; follicular lymphoma	Bristol–Myers Squibb
AMD11070/AMD070	Phase 1/phase 2	HIV infections	National Institute of Allergy and Infectious Diseases (NIAID); AIDS Clinical Trials Group
MSX-122	Phase 1	Solid tumors	Metastatix, Inc.
POL6326	Phase 1	Mobilization of hematopoietic stem cells in healthy volunteers	Polyphor Ltd.
AMD070	Phase 1	HIV infections	National Institute of Allergy and Infectious Diseases (NIAID); AIDS Clinical Trials Group

In addition to ongoing clinical trials, several different CXCR4 antagonists are under investigation in *in vitro* and *in vivo* experimental research. For example, TG-0054 has recently been investigated for its therapeutic potential in an animal model of myocardial infarction. It could be shown that the administration of TG-0054 after induction of myocardial infarction leads to a mobilization of mesenchymal stem cells, preventing left ventricular dysfunction and causing a decrease in inflammatory cytokine levels (246). A phase II clinical trial to assess the pharmacokinetics and safety of TG-0054 for the mobilization of stem cells in patients with multiple myeloma, non-Hodgkin lymphoma, or Hodgkin disease has been completed by the end of 2014; however, the results have not been published to date (clinical trials NCT01458288).

Since AMD3100 is currently the only FDA-approved CXCR4 antagonist, it is being used in multiple clinical trials (search term “AMD3100” at clinicaltrials.gov reveals 112 hits). Additionally, researchers are constantly aiming to improve AMD3100, for example, efforts to increase its anti-HIV properties by functionalizing the phenyl moiety are still ongoing (233). Mechanistically, AMD3100 prevents the binding of CXCL12 to CXCR4 and thus CXCR4 downstream signaling. AMD3465, an analog of AMD3100, similarly prevents CXCL12 binding to CXCR4 with an even 10-fold higher effectiveness (247), however did not yet receive FDA approval. Of note, AMD3100 was previously reported to be able to enhance CXCL12 binding to ACKR3 and to induce basal as well as CXCL12-induced β-arrestin recruitment to ACKR3 above concentrations of 10 μM, warranting AMD3100 not only to interfere with CXCR4 signaling but also to induce ACKR3 signaling at high concentrations or modulate CXCL12 scavenging through ACKR3 (248).

## Concluding Remarks

In the last decade, many new insights in the CXCR4 signaling cascade have been revealed, both in terms of the ligands to which CXCR4 binds, the different signaling pathways that are initiated downstream, the formation of heteromeric receptor complexes involving CXCR4, as well as the interaction of other receptor complexes with the CXCR4 cascade. This will considerably help to understand the functions of CXCR4 and its ligands both in physiological settings as well as in disease. However, there is still a lot of haziness that remains to be clarified. For example, the specific residues of CXCR4 involved in the site 1 and site 2 interaction with CXCL12 vs. MIF are still unknown, as is its affinity to CXCL12 compared to MIF binding in physiological settings, and thus the potential competition between CXCL12 and MIF for CXCR4 binding. Furthermore, both CXCL12 and MIF can interact with ACKR3, and, despite the initial view that ACKR3 merely serves as a chemokine decoy receptor, first reports have now indicated that both MIF- and CXCL12-induced ACKR3 signaling regulate important biological processes, and it can be expected that additional functions of these signaling pathways in both physiological as well as pathological conditions will be revealed. Also, the roles of CXCR4, CXCL12, and MIF in certain physiological or pathophysiological settings may be very complex depending on the cell type, microenvironment, and perhaps potentially inter-individual settings. For example, CXCR4, CXCL12, as well as MIF have been associated with a pro- as well as anti-inflammatory role after myocardial infarction (98, 99, 239–242), and also in the context of injury-induced restenosis, CXCR4 has been associated with detrimental as well as protective effects (249, 250), as discussed in more detail recently by Döring et al. (1). Based on the fact that CXCR4 can interact with ACKR3 and CD74, but also other receptors as TLR2, it could be speculated that these double-edged functions may result from the involvement of differential receptor complexes that initiate different intracellular signaling pathways. Revealing differential interaction sites for CXCR4 with CXCL12 vs. MIF, and differential signaling pathways that mediate pro- vs. inflammatory properties could reveal new strategies to target only specific aspects of the CXCR4 signaling cascade, while leaving others unaffected to prevent unwanted side effects.

## Conflict of Interest Statement

The authors declare that the research was conducted in the absence of any commercial or financial relationships that could be construed as a potential conflict of interest.
